# Detection of placental stiffness using virtual magnetic resonance elastography in pregnancies complicated by preeclampsia

**DOI:** 10.1007/s00404-024-07585-0

**Published:** 2024-06-17

**Authors:** Jialu Xu, Yajing Mao, Feifei Qu, Xiaolin Hua, Jiejun Cheng

**Affiliations:** 1 Department of Radiology, Shanghai First Maternity and Infant Hospital, School of Medicine, Tongji University, Shanghai, 200092 China; 2 Department of Obstetrics, Shanghai First Maternity and Infant Hospital, School of Medicine, Tongji University, Shanghai, 200092 China; 3 MR Collaboration, Siemens Healthineers Ltd, Shanghai, China

## Introduction

Preeclampsia (PE) is a multisystem disorder of pregnancy affecting 3–5% of pregnancies [1]. It is a major cause of perinatal morbidity and mortality [2]. PE is defined by the International Society for the Study of Hypertension in Pregnancy (ISSHP) as the occurrence of hypertension that develops after the 20th week of pregnancy, accompanied by proteinuria or signs of acute kidney injury, liver dysfunction, neurological manifestations, hemolysis, thrombocytopenia, or fetal growth restriction (FGR) [3]. The maternal vascular malperfusion in PE is characterized histologically by diminished placental size, infarction, abnormal development of the placental villi, and a deficiency of the maternal decidual spiral arterioles [4, 5]. Although the cause of PE is unknown, the accumulated evidence strongly implicates the placenta [6, 7].

As PE is closely related to placental dysfunction, the antenatal assessment of placental function is crucial to understand this complex condition [8]. Placental imaging offers a window into the placental contribution and mechanisms. Studies have reported that the mean placental stiffness detected using ultrasound is considerably higher in preeclamptic pregnancies than in controls in the third trimester [9]. The elasticity of the in vivo placentas used to be detected by ultrasound shear wave elastography (SWE) because magnetic resonance elastography is not recommended for pregnant patients. However, the accuracy of SWE measurement depends on the technique and experience of the operator. Moreover, the posterior placenta is hard to obtain adequate measurements.

In this study, we employed virtual magnetic resonance elastography (vMRE) for assessing placental stiffness. The vMRE used a diffusion-weighted imaging (DWI) sequence to enhance the texture of tissues with dense diffusion barriers, such as fibrotic tissues. Le Bihan et al. proposed a DWI-based vMRE to evaluate liver fibrosis. They demonstrated a remarkable correlation between the DWI-based shear modulus (*μ*_diff_) and the MRE shear modulus (μMRE) [10]. A few studies have suggested that vMRE can be used to measure the stiffness of soft tissues [11–16]. In recent years, this approach has also been used in studies on the placenta [17]. However, the value of vMRE for detecting the placental stiffness of pregnancies with PE is still unknown. Therefore, this study aimed to explore the value of DWI-based elasticity in pregnancies with PE.

## Materials and methods

### Study population

This prospective case–control study was approved by the institutional ethics committee in our hospital (Ethics approval number: KS23253). Seventy-four PE pregnancies and eighty-two control pregnancies participated in this study were recruited from January 2023 to September 2023. We successfully obtained the informed written consent from each participant. The inclusion criteria were as follows: (1) the maternal age ≥ 20 years old; (2) singleton pregnancies of 20 weeks gestation or later, (3) Preeclampsia was prospectively defined using the international consensus definition: gestational hypertension accompanied by one or more of the following new-onset conditions at or after 20 weeks’ gestation: proteinuria, acute kidney injury, liver involvement, neurological complications, hematological complications, and uteroplacental dysfunction [18]. The normal control group of pregnancies consisted of healthy women with normal ultrasound results and birthweight. They underwent MRI during 21–35 weeks’ gestation.

**Fig. 1:**
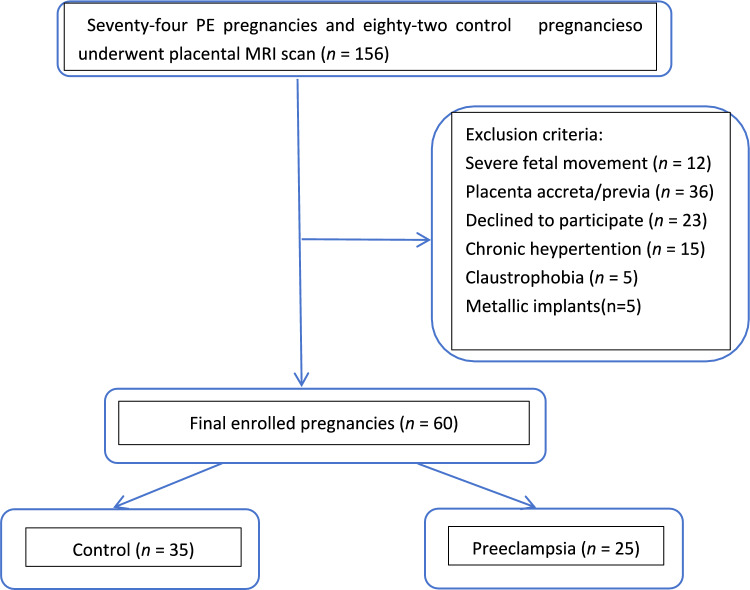
Flow diagram of patient recruitment of the study

The exclusion criteria were as follows: (1) poor-quality MRI images with severe fetal movement; (2) chronic hypertension; (3) placental abnormalities including placental implantation, placental previa, and placental abruption; (4) metallic implants; and (5) claustrophobia. The control group of pregnant women when the following criteria were fulfilled: singleton pregnancies of 20 weeks gestation or later, no diagnosis of a hypertensive disorder at enrollment and until delivery, no significant past medical history, no pregnancy complications (including gestational diabetes mellitus), delivery at term with birth weight between the 3rd and 97th centiles (calculated using International Fetal and Newborn Growth Consortium for the 21st Century version 1.3.5). A total of 60 pregnant women were enrolled in the final analysis, including 35 controls and 25 pregnant women with PE (Fig. 1). vMRE measurements were obtained upon diagnosis of PE in the outpatient or inpatient setting. All patients received routine care for managing PE, which was based on established protocols and the clinical expertise of the supervising obstetrician. This included expectant management, treatment of hypertensive emergencies with anti-hypertensive medications, and use of magnesium sulfate for seizure prophylaxis or delivery when deemed necessary.

## Magnetic resonance imaging protocol

All patients were examined on a 1.5-T scanner (MAGNETOM Aera, Siemens Healthineers AG, Erlangen, Germany). The magnetic resonance imaging (MRI) protocols were as follows: T1-weighted VIBE sequence [repetition time (TR)/echo time (TE) = 4.85 ms/2.38 ms slice thickness = 5.0 mm; field of view (FOV) = 400 × 368 mm^2^; flip angle (FA) = 10°; and in-plane resolution = 1.5 × 1.5 mm^2^]; T2-weighted half-Fourier-acquired single-shot turbo spin echo sequence (TR/TE = 1300 ms/109 ms; slice thickness = 5.0 mm; FOV = 400 × 309 mm^2^; FA = 120°, and in-plane resolution = 1.5 × 1.5 mm^2^); and multi-*b* value DWI sequence (TR/TE = 6400 ms/65 ms; slice thickness = 5 mm; FOV = 320 × 320 mm^2^; and in-plane resolution = 3.5 × 3.5 mm^2^) with a spectrum of varying *b* values of 50, 200, and 800 s/mm^2^. The total scan duration was less than 10 min.

## Measurement of placental stiffness

The region of interest (ROI) segmentation was performed using the open-source software ITK-SNAP [18]. The stiffness value of DWI-based vMRE was determined using custom-written software in MATLAB (MathWorks, MA, USA). DWI of the lower *b* value (*S*_low_, *b* value = 200 s/mm^2^) and that of the higher *b* value (*S*_high_, *b* value = 800 s/mm^2^) were used to estimate the virtual stiffness presented by the *μ*_diff_: *µ*_diff_ = *α*·(ln *S*_*low*_/*S*_*high*_) + *β*

where *α* is the scaling factor and *β* is the shift factor, set to − 9.8 and 14, respectively, according to the previous calibration studies on the liver [19]. The mean values of placental *μ*_diff_ were automatically extracted from the segmented placenta regions using the custom-written software.

## Statistical analysis

The statistical analyses were performed using SPSS software (version 27.0). The Student *t* test was used to perform pairwise comparisons. The *χ*^2^ test was used to test the association between categorical variables. Multiple regression analysis was used to assess the relationship between placental stiffness and PE. The receiver-operating characteristic (ROC) curve analysis and the area under the curve (AUC) were used to quantify and compare the diagnostic value of each remarkable parameter. The potential relationship between *μ*_diff_ and gestational week at MRI in normal pregnancies was explored using a linear regression model. A *P* value < 0.05 indicated a statistically significant difference.

## Results

### Baseline characteristics

The details regarding the baseline and clinical characteristics of the participants are summarized in Table 1. No substantial differences in the mean maternal age, body mass index (BMI) at admission, or mean gestational age (GA) at the time of MRI scanning were noted between the control and PE groups. The PE group was associated with a significantly increased rate of preterm delivery (52 vs 14.3%; *P* = 0.009) and reduced final birthweight (2138.57 ± 837 g vs 3299.10 ± 539 g; *P* = 0.014) (Table 1).

**Table 1:** Baseline of the control group (*n* = 35) and the PE group (*n* = 25)

	Control (*n* = 35)	PE (*n* = 25)	*P*
Maternal age (years)	33.16 ± 3.82	33.07 ± 3.45	.351
BMI at delivery	21.82 ± 3.31	23.036 ± 3.50	.175
GA at the time of MRI scan (weeks)	33 ± 5	33 ± 2	.463
GA at delivery (weeks)	38 ± 4	37 ± 3	.232
Preterm deliveries	5 (14.3%)	13 (52%)	.009
*Routes of delivery*			.048
Vaginal delivery	19 (35)	2 (25)	
Cesarean section	16 (35)	23 (25)	
Male	18 (35)	12 (25)	
Female	17 (35)	13 (25)	
1-min Apgar score	10	9.3	.762
Birthweight (g)	3299.10 ± 539	2138.57 ± 837	.014

*BMI* Body mass index, *GA* gestational age, *MRI* magnetic resonance imaging, *PE* preeclampsia

### Comparison of ADC and *μ*_diff_ between control and PE groups

**Fig. 2:**
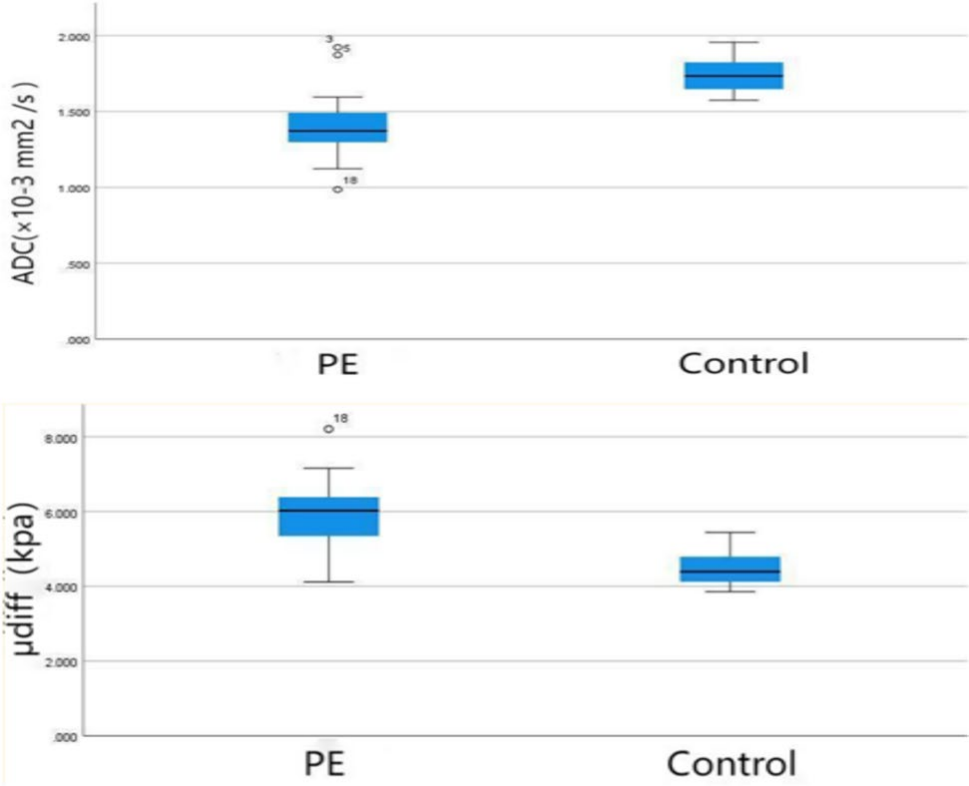
Box and whisker plots of the placental ADC and *μ*_diff_ values in the control and PE groups. ADC; *μ*_diff_ DWI-based shear modulus, *PE* preeclampsia

**Fig. 3:**
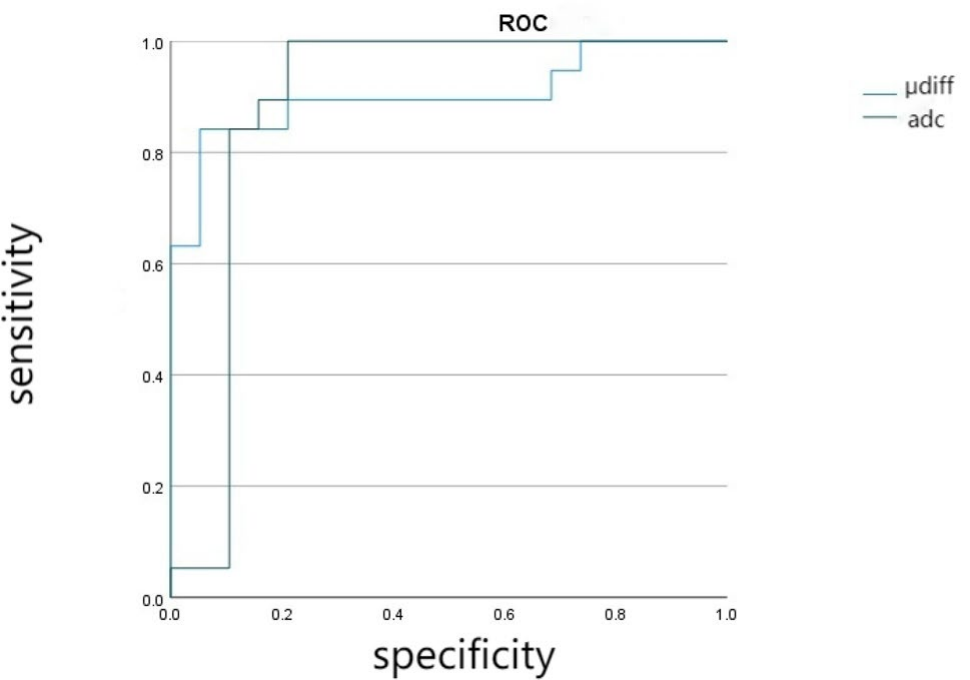
ROC curves of the *μ*_diff_ and ADC values. ADC; *μ*_diff_ DWI-based shear modulus, *ROC* receiver-operating characteristic

**Fig. 4:**
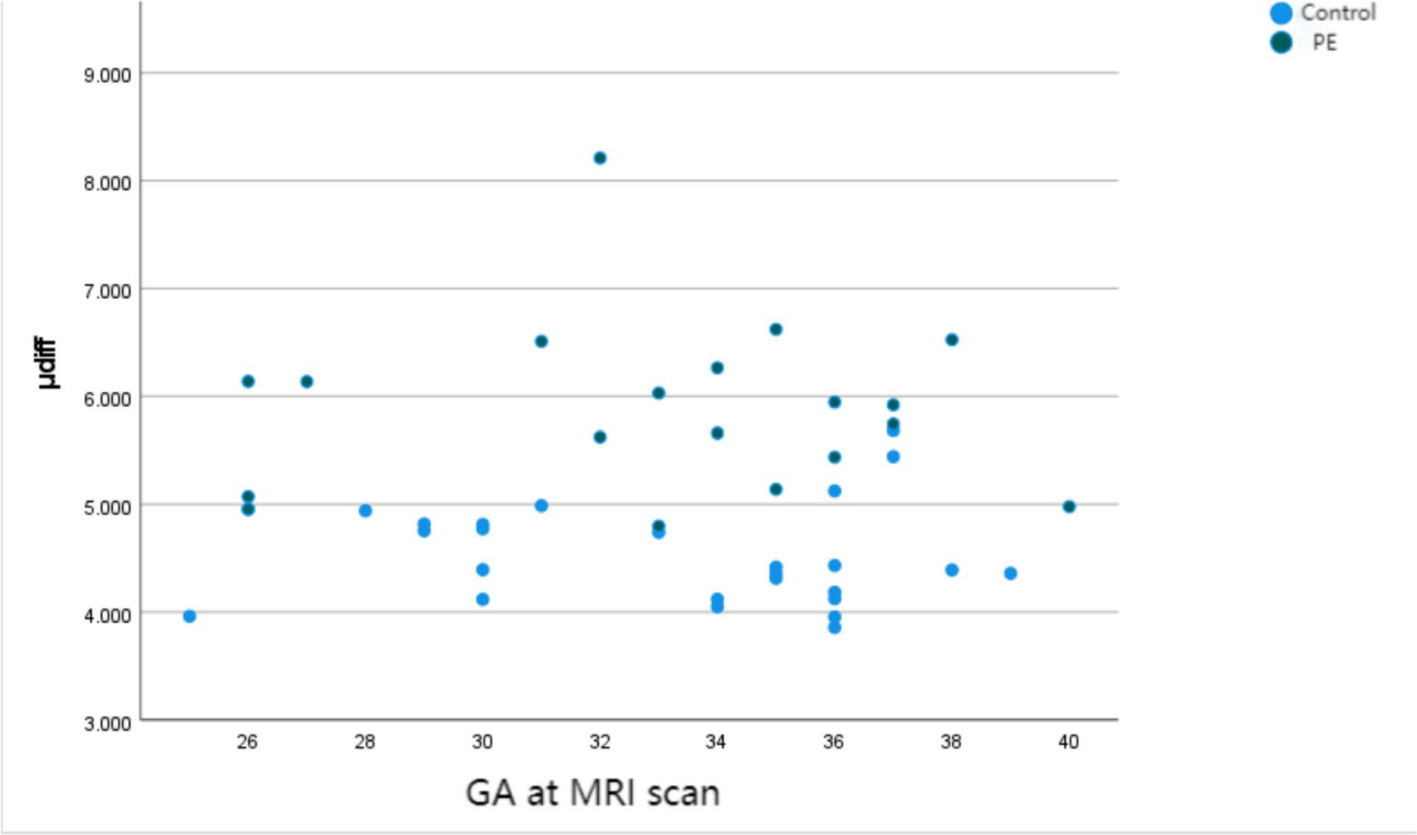
Placental stiffness measurement distribution at varying gestational ages in the control and PE groups. GA at MRI scan (week); *μ*_diff_, DWI-based shear modulus, *PE* preeclampsia

The mean ADC value was lower in the PE group compared with the control group (1.414 ± 0.228 × 10^–3^ mm^2^/s vs 1.737 ± 0.107 × 10^–3^ mm^2^/s, *P* = 0.031) (Fig. 2; Table 2). The mean μ_diff_ value was 5.901 ± 1.757 kPa and 4.618 ± 2.055 kPa for the PE and control groups, respectively (Fig. 2; Table 2). The AUC of the ROC curve was 0.903 and 0.796 for the PE and control groups, respectively (Fig. 3; Table 2). The optimized cut-off value of placental stiffness value for the presence of PE was 4.95 kPa. The variates such as GA, BMI and maternal age did not reach statistical significance as predictors of the placental stiffness value. (Fig. 4; Table 3).

**Table 2:** Comparison of relevant parameters in the normal and PE groups

Parameter	AUC (95% CI)	Mean value control group (*n* = 35)	PE group (*n* = 25)	*P* value
*μ*_diff_ (kPa)	0.903 (0.797–1.000)	4.618 ± 2.055	5.901 ± 1.757	.011
ADC (10^–3^/mm^2^)	0.796 (0.734–1.000)	1.737 ± 0.107	1.414 ± 0.228	.031

*ADC AUC* area under the curve, *CI* confidence interval, *μ*_diff_ DWI-based shear modulus, *PE* preeclampsia

**Table 3:** Effect of PE and common factors on placental stiffness measured by vMRE (*μ*_diff_) calculated using multiple regression analysis

	*B*	Standard error	*β*	*t*	*P* value
Parameter	10.028	1.050		9.554	.000
PE	1.263	.197	.705	6.396	.000
BMI	.018	.022	.065	.835	.410
Age	.006	.020	.022	.284	.778
GA	.046	.044	.193	1.065	.294

*BMI* Body mass index, *μ*_diff_ DWI-based shear modulus, *PE* preeclampsia, *vMRE* virtual magnetic resonance elastography

### Correlation between placental stiffness and perinatal outcomes

We grouped the study population based on the optimized cut-off value for placental stiffness (e.g., Group A = placental stiffness < 4.95 kPa; Group B = placental stiffness ≥ 4.95 kPa) and evaluated their respective pregnancies and neonatal outcomes. We observed that Group B had substantially lower birth weight and GA at the time of delivery (2340 ± 958 g and 34.92 ± 4.18 weeks, respectively) compared Group A (3265 ± 580 g and 38.11 ± 1.52 weeks, respectively) (Table 4).

**Table 4:** Neonatal outcomes using optimized cut-off value of placental stiffness

	Group A (*μ*_diff_ < 4.95 kPa)	Group B (*μ*_diff_ ≥ 4.95 kPa)	*P* value
BMI	21.86 ± 3.58	22.83 ± 3.8	.320
Age (years)	33.37 ± 3.83	32.29 ± 4.07	.687
GA at delivery (weeks)	38.11 ± 1.52	34.92 ± 4.18	.037
Birth weight (g)	3265 ± 580	2340 ± 958	.002

*GA* Gestational age, *μ*_diff_ DWI-based shear modulus

## Discussion

In this study, we determined that the mean placental stiffness was remarkably higher in PE pregnancies than in controls, and was not affected by maternal age or GA, which was in line with previous findings [19]. However, contrasting perspectives exist in the literature; for instance, Liu et al. observed that the placental stiffness value was the lowest in 26 weeks and exhibited an upward trend from 26 to 36 weeks [20]. PE is described as an excessive maternal inflammatory response to a dysfunctional placenta or the vascular load of pregnancy itself. In normal pregnancy, placentation occurs by trophoblast invasion of the maternal spiral arteries, resulting in low-resistance, high-flow maternal uteroplacental circulation [21, 22]. In PE pregnancies, trophoblast invasion of the maternal spiral arteries is impaired, resulting in reduced placental perfusion, which creates a hypoxic environment in the placenta [21, 23]. Hypoxia stimulates collagen deposition, vascular fibrin deposition, and fibrosis, leading to higher tissue stiffness [24]. PE placentas exhibit injuries such as placental vascular lesions and vesicular fibrosis, syncytial knots and microcalcifications, and perivillous fibrin deposition and villous infarcts. These findings explain the increased placental stiffness seen in PE [25–27].

ADC has been reported to be lower in abnormal placentas than in normal placentas. Restricted diffusion may result from reduced gas exchange area in PE-complicated placentas [28, 29]. The present study suggested that the ADC value was remarkably lower in PE placentas than in normal placentas. The ROC curves of ADC and *μ*_diff_ values were compared. The results revealed that* μ*_diff_ imaging performed better than ADC in PE placentas. Accordingly, compared with ADC, *μ*_diff_ is seemingly more accurate than DWI sequences in assessing dysfunctional placentas in clinical practice. Despite the widespread use of ADC, its real status of diffusivity may be limited by the monoexponential model because other factors, such as microcirculation and diffusion of water molecules, can easily interfere with it [30]. Intravoxel incoherent motion(IVIM) sequences have been developed to overcome these disadvantages using multiple b values, but the acquisition time is considerably prolonged [31, 32].

In addition, we observed that neither GA nor maternal age had noteworthy correlation with the μ_diff_ value at MRI, consistent with the previous researches [9, 17, 33]. However, Spiliopoulos M et al. found that BMI was the only statistically significant predictor of the model with stiffness decreasing with higher BMI (inverse correlation) [9]. In their research, the BMI of pregnancies was 32.5 ± 1.4 and 34.7 ± 1.6 in the control and PE group. While in our study, the BMI of pregnancies was 21.8 ± 3.3 and 23.0 ± 3.5 in the control and PE group. BMI (calculated as weight in kilograms divided by height in meters squared) was classified in the WHO definition (underweight, < 18.5; reference weight, 18.5–24.9; overweight, 25.0–29.9; obese, ≥ 30.0) [34]. In China, pre-pregnancy BMI was categorized using the Chinese cutoffs, which are slightly different than the WHO definition (underweight, < 18.5; reference weight, 18.5–23.9; overweight, 24–27.9; obese, ≥ 28) [35].The contradiction between our results could be attributed to the individual differences and the study population selection.

The limitation to this work is that the relationship between *μ*_diff_ and GA needs to be further investigated with a larger sample size. Studies on placental elastography are limited. Therefore, whether increased stiffness is seen in only PE or may be present with other causes of placental insufficiency, such as chronic hypertension, is unknown.

## Conclusions

Compared with healthy pregnancies, placentas of PE pregnancies are stiffer. Placental vMRE was demonstrated to be more reliable than ADC in differentiating between normal and PE placentas. Placental stiffness is not affected by GA. It is also more likely to be associated with poor perinatal outcomes such as lower birth weight and earlier GA at delivery.

## Data Availability

The institutional ethics committee in our hospital (Ethics approval number: KS23253).

## References

[1] Chappell LC, Cluver CA, Kingdom J, Tong S (2021) Pre-eclampsia. Lancet 398(10297):341–354 34051884 10.1016/S0140-6736(20)32335-7

[2] Say L, Chou D, Gemmill A et al (2014) Global causes of maternal death: a WHO systematic analysis. Lancet Glob Health 2(6):e323–e333 25103301 10.1016/S2214-109X(14)70227-X

[3] Brown MA, Magee LA, Kenny LC et al (2018) Hypertensive disorders of pregnancy. Hypertension 72(1):24–43 29899139 10.1161/HYPERTENSIONAHA.117.10803

[4] Wright E, Audette MC, Ye XY et al (2017) Maternal vascular malperfusion and adverse perinatal outcomes in low-risk nulliparous women. Obstet Gynecol 130(5):1112–1120 29016509 10.1097/AOG.0000000000002264

[5] Khong TY, Mooney EE, Ariel I et al (2016) Sampling and definitions of placental lesions: amsterdam placental workshop group consensus statement. Arch Pathol Lab Med 140(7):698–713 27223167 10.5858/arpa.2015-0225-CC

[6] Fisher SJ (2015) Why is placentation abnormal in preeclampsia. Am J Obstet Gynecol 213(4):S115-22 26428489 10.1016/j.ajog.2015.08.042 PMC4592742

[7] Liu XF, Lu JJ, Li MD, Li Y, Zeng AR, Qiang JW (2022) Prediction of pre-eclampsia by using radiomics nomogram from gestational hypertension patients. Front Neurosci 16:961348 35992933 10.3389/fnins.2022.961348 PMC9389207

[8] Lagerstrand K, Gaedes N, Eriksson S et al (2021) Virtual magnetic resonance elastography has the feasibility to evaluate preoperative pituitary adenoma consistency. Pituitary 24(4):530–541 33555485 10.1007/s11102-021-01129-4 PMC8270838

[9] Spiliopoulos M, Kuo CY, Eranki A et al (2020) Characterizing placental stiffness using ultrasound shear-wave elastography in healthy and preeclamptic pregnancies. Arch Gynecol Obstet 302:1103–1112 32676857 10.1007/s00404-020-05697-x PMC7646518

[10] Le Bihan D, Ichikawa S, Motosugi U (2017) Diffusion and intravoxel incoherent motion MR imaging-based virtual elastography: a hypothesis generating study in the liver. Radiology 285(2):609–619 28604279 10.1148/radiol.2017170025

[11] Abu AN, Dillman JR, Gandhi DB, Dudley JA, Trout AT, Miethke AG (2021) Association between liver diffusion-weighted imaging apparent difusioncoefcient values and other measures of liver disease in pediatric autoimmune liver disease patients. Abdom Radiol 46(1):197–204 10.1007/s00261-020-02595-3 PMC8530174 32462385

[12] Aunan-Diop JS, Andersen M, Friismose AI et al (2023) Virtual magnetic resonance elastography predicts the intraoperative consistency of meningiomas. J Neuroradiol 50(4):396–401 (**[PubMed:36343849]**) 36343849 10.1016/j.neurad.2022.10.006

[13] Jung HN, Ryoo I, Suh S, Lee YH (2024) Kim E (2024) Evaluating the elasticity of metastatic cervical lymph nodes in head and neck squamous cell carcinoma patients using DWI-based virtual MR elastography. Magn Reson Med Sci 23(1):49–55 36529497 10.2463/mrms.mp.2022-0082 PMC10838712

[14] Kromrey ML, Le Bihan D, Ichikawa S, Motosugi U (2020) Diffusion weighted MRI-based virtual elastography for the assessment of liver fbrosis. Radiology 295(1):127–135 32043948 10.1148/radiol.2020191498

[15] Lagerstrand K, Gaedes N, Eriksson S et al (2021) Virtual magnetic resonance elastography has the feasibility to evaluate preoperative pituitary adenoma consistency. Pituitary 24(4):530–541 33555485 10.1007/s11102-021-01129-4 PMC8270838

[16] Yin Z, Magin RL, Klatt D (2014) Simultaneous MR elastography and diffusion acquisitions: diffusion-MRE (dMRE). Magn Reson Med 71(5):1682–8 24648402 10.1002/mrm.25180

[17] Deng J, Cao Y, Yao L et al (2023) Value of placental virtual magnetic resonance elastography and intravoxel incoherent motion-based diffusion and perfusion in predicting adverse outcomes of small-for-gestational-age infants. Insights Imaging 14(1):153 37741945 10.1186/s13244-023-01503-9 PMC10517907

[18] Yushkevich PA, Piven J, Hazlett HC et al (2006) User-guided 3D active contour segmentation of anatomical structures: significantly improved efficiency and reliability. Neuroimage 31(3):1116–28 16545965 10.1016/j.neuroimage.2006.01.015

[19] Brown MA, Magee Laura A, Kenny Louise C, Karumanchi SA, McCarthy PF, Saito S et al (2018) Hypertensive disorders of pregnancy: ISSHP classification diagnosis & management recommendations for international practice. Pregnancy Hypertens 13:291–310 29803330 10.1016/j.preghy.2018.05.004

[20] Sørensen A, Sinding M (2020) Preeclamptic placenta new insights using placental magnetic resonance imaging. Hypertension 75(6):1412–1413 32401645 10.1161/HYPERTENSIONAHA.120.14855

[21] Freedman BR, Bade ND, Riggin CN, Zhang S, Haines PG, Ong KL et al (2015) The (dys) functional extracellular matrix. Biochim Biophys Acta. 1853(11):3153–64 25930943 10.1016/j.bbamcr.2015.04.015 PMC4846286

[22] Gilkes DM, Bajpai S, Chaturvedi P, Wirtz D, Semenza GL (2013) Hypoxia-inducible factor 1 (HIF-1) promotes extracellular matrix remodeling under hypoxic conditions by inducing P4HA1, P4HA2, and PLOD2 expression in fibroblasts. J Biol Chem. 288(15):10819–29 23423382 10.1074/jbc.M112.442939 PMC3624462

[23] Ghidini A, Salafa CM, Pezzullo JC (1997) Placental vascular lesions and likelihood of diagnosis of preeclampsia. Obstet Gynecol 90(4 Pt 1):542–5 9380313 10.1016/S0029-7844(97)00360-8

[24] Nikitina ER, Mikhailov AV, Nikandrova ES, Frolova EV, Fadeev AV, Shman VV et al (2011) In preeclampsia endogenous cardiotonic steroids induce vascular fbrosis and impair relaxation of umbilical arteries. J Hypertens. 29(4):769–776 21330936 10.1097/HJH.0b013e32834436a7 PMC3053428

[25] Orabona R, Donzelli CM, Falchetti M, Santoro A, Valcamonico A, Frusca T (2016) Placental histological patterns and uterine artery Doppler velocimetry in pregnancies complicated by early or late pre-eclampsia. Ultrasound Obstet Gynecol 47(5):580–585 26511592 10.1002/uog.15799

[26] Ho A, JanaHutter PS et al (2021) Placental magnetic resonance imaging in chronic hypertension: a case-control study. Placenta 1(104):138–145 10.1016/j.placenta.2020.12.006 PMC7921773 33341490

[27] Malayeri AA, El Khouli RH, Zaheer A et al (2011) Principles and applications of diffusion-weighted imaging in cancer detection, staging, and treatment follow-up. Radiographics 31(6):1773–1791 21997994 10.1148/rg.316115515 PMC8996338

[28] Gorkem SB, Coskun A, Eslik M, Kutuk MS, Ozturk A (2019) Diffusion-weighted imaging of placenta in intrauterine growth restriction with worsening Doppler US findings. Diagn Interv Radiol 25(4):280–285 31120426 10.5152/dir.2019.18358 PMC6622434

[29] He J, Chen Z, Chen C, Liu P (2023) Differences in placental oxygenation and perfusion status between fetal growth-restricted and small-for-gestational-age pregnancies: a functional magnetic resonance imaging study. Eur Radiol 33(3):1729–1736 36269372 10.1007/s00330-022-09185-5

[30] Chaiworapongsa T, Chaemsaithong P, Yeo L, Romero R (2014) Pre-eclampsia part 1: current understanding of its pathophysiology. Nat Rev Nephrol 10(8):466–480 25003615 10.1038/nrneph.2014.102 PMC5893150

[31] Burton GJ (2009) Oxygen, the Janus gas; its effects on human placental development and function. J Anat 215(1):27–35 19175804 10.1111/j.1469-7580.2008.00978.x PMC2714636

[32] Meng N, Fang T, Feng P et al (2021) Amide proton transfer-weighted imaging and multiple models diffusion-weighted imaging facilitates preoperative risk stratifcation of early-stage endometrial carcinoma. J Magn Reson Imaging 54(4):1200–1211 33991377 10.1002/jmri.27684

[33] Lai HW, Lyv GR, Wei YT, Zhou T (2020) The diagnostic value of two-dimensional shear wave elastography in gestational diabetes mellitus. Placenta 101:147–153 32980791 10.1016/j.placenta.2020.08.024

[34] Yang Y, Isabelle LR, Zhu J et al (2021) Preeclampsia prevalence, risk factors, and pregnancy outcomes in Sweden and China. JAMA Netw Open 4(5):e218401 33970258 10.1001/jamanetworkopen.2021.8401 PMC8111481

[35] He W, Li Q, Yang M et al (2015) Lower BMI cutoffs to define overweight and obesity in China. Obesity (Silver Spring) 23(3):684–691 25645003 10.1002/oby.20995

