# Correction: Detection of placental stiffness using virtual magnetic resonance elastography in pregnancies complicated by preeclampsia

**DOI:** 10.1007/s00404-024-07645-5

**Published:** 2024-07-24

**Authors:** Jialu Xu, Yajing Mao, Feifei Qu, Xiaolin Hua, Jiejun Cheng

**Affiliations:** 1 Department of Radiology, Shanghai First Maternity and Infant Hospital, School of Medicine, Tongji University, Shanghai, 201204 China; 2 Department of Obstetrics, Shanghai First Maternity and Infant Hospital, School of Medicine, Tongji University, Shanghai, 201204 China; 3 MR Collaboration, Siemens Healthineers Ltd, Shanghai, China

**Correction: Archives of Gynecology and Obstetrics** 10.1007/s00404-024-07585-0

In this article, the author Jiejun Cheng and Xiaolin Hua are joint corresponding author. Jialu Xu and Yajing Mao are joint first authors.

The original article has been corrected.

